# Precision prescribing of SGLT2-inhibitors in people with type 2 diabetes for primary prevention of heart failure: model development and validation study

**DOI:** 10.2337/dc25-2973

**Published:** 2026-06-01

**Authors:** Katherine G Young, Andrew P McGovern, Rhian Hopkins, Thijs T Jansz, Pedro M Cardoso, Rury R Holman, Ewan R Pearson, Andrew T Hattersley, Angus G Jones, Kieran Docherty, Naveed Sattar, Beverley M Shields, John M Dennis

**Affiliations:** 1 Clinical and Biomedical Sciences, University of Exeter Medical School, Exeter, UK; 2 Diabetes Trials Unit, Radcliffe Department of Medicine, University of Oxford, Oxford, UK; 3 Division of Diabetes, Endocrinology and Reproductive Medicine, Ninewells Hospital and Medical School, University of Dundee, Dundee, UK; 4 School of Cardiovascular and Metabolic Health, University of Glasgow, Glasgow, UK

## Abstract

**Objective:**

SGLT2-inhibitors (SGLT2i) reduce heart failure (HF) risk in type 2 diabetes (T2D) and are recommended for T2D patients with atherosclerotic cardiovascular disease (ASCVD), HF or chronic kidney disease (CKD). However, most people with T2D do not have these conditions, and current guidelines for this group do not indicate which individuals may benefit most from SGLT2i. We aimed to develop and validate a model to predict individual-level HF benefit of SGLT2i in people with T2D without ASCVD/HF/CKD.

**Research Design and Methods:**

We developed the SGLT2-inhibitor Absolute Benefit REsponse (SABRE) model, combining absolute HF risk from the validated QDiabetes-HF model with the SGLT2i -associated hazard ratio for HF hospitalisation from trial meta-analysis (HR 0.63) to estimate individual 5-year HF benefit. Model components and predictions were validated using UK primary care data with linked hospital and death records from 2013-2020.

**Results:**

Among 57,368 SGLT2i initiators and 111,673 comparator (DPP4-inhibitor/sulfonylurea) initiators, SGLT2i use was associated with a 30% lower risk of new-onset HF (HR 0.70 [95% CI 0.63–0.78]), consistent with trial evidence. Relative HF benefit did not vary by baseline absolute heart failure risk (p=0.82). SABRE model predicted 5-year absolute HF benefit with SGLT2i ranged from <0.1% to 14.1 % (median 1.0% [IQR 0.6-1.8%]) and calibrated well against observed HF outcomes. SABRE provided more targeted HF prevention than current guidelines in those with T2D without ASCVD/HF/CKD.

**Conclusions:**

The SABRE model is an easily deployed clinical prediction model integrating trial evidence and allowing more precise targeting of SGLT2-inhibitors for primary HF prevention in T2D.

## Introduction

SGLT2-inhibitors (SGLT2i) substantially reduce the risk of hospitalisation for heart failure and, to a lesser extent, reduce atherosclerotic cardiovascular disease (ASCVD) events in people with type 2 diabetes (T2D) (1). Guidelines consistently recommend SGLT2i for individuals with T2D with established ASCVD or history of heart failure (HF) (2; 3), who have the highest absolute risk of both ASCVD events and heart failure and are therefore likely to derive the greatest benefit. However, in those with T2D without ASCVD or HF, evidence from trial meta-analysis of SGLT2i-associated ASCVD benefit is uncertain (1). This group represents around two-thirds of those with T2D in the UK (4).

For those with T2D without ASCVD/HF, international (ADA/EASD) guidance suggests the use of SGLT2i in those aged ≥55 years with the presence of 2 or more established cardiovascular risk factors including obesity, hypertension, or dyslipidaemia (2). In contrast, UK guidance for SGLT2i targeting in this group is now based on an individual-level predicted 10-year ASCVD risk of ≥10% (3), a threshold exceeded by 89.6% of people with T2D without ASCVD/HF in the UK on anti-hyperglycaemic treatment (4). Beyond these broad recommendations, current guidance does not give advice regarding which people with T2D without ASCVD/HF are likely to have the greatest cardiovascular benefit if treated with SGLT2i.

We aimed to develop and validate an ‘SGLT2i Absolute Benefit REsponse’ model (SABRE) to predict the absolute benefit of SGLT2i for primary prevention of heart failure for individual patients with T2D without ASCVD/HF. We followed the risk-modelling approach outlined in the PATH (Predictive Approaches to Treatment effect Heterogeneity) statement (5). Individual-level predictions were derived by combining the RCT-estimated relative risk reduction of SGLT2i for heart failure prevention, and absolute heart failure risk estimates from an established validated clinical prediction model (6). Accuracy and clinical utility were assessed in a large population-representative UK dataset of people with T2D.

## Research Design and Methods

### Study population

We used longitudinal UK primary care data from Clinical Practice Research Datalink (CPRD) Aurum (7) with linkage to national hospital inpatient data (Hospital Episode Statistics), Office for National Statistics death data and patient Index of Multiple Deprivation (IMD) data. CPRD is population-representative and covers around 20% of the UK population (7; 8).

The study population was those with type 2 diabetes (T2D), without atherosclerotic cardiovascular disease (ASCVD), heart failure, or chronic kidney disease (CKD; stage 3a–5 or macroalbuminuria), i.e. excluding those for whom evidence-based guidance for SGLT2i treatment already exists. We included those initiating SGLT2i, or as a comparator, those initiating DPP4-inhibitors (DPP4i) or sulfonylureas (SU) and not taking SGLT2i, between 1 January 2013 and 31 October 2020. Treatment initiation was defined as the first-ever prescription for each drug class, defining start of follow-up. Further exclusions were those receiving a GLP1-receptor agonist or thiazolidinedione as these treatments may modify heart failure risk (9; 10), those aged<25 or >84 years, those prescribed SGLT2i, DPP4i or SU as first-line treatment (not recommended in UK guidelines) (3), and drug initiations within 90 days of GP registration (which may represent continuation of treatment). Codelists and implementation algorithms for study variables are described here: https://github.com/Exeter-Diabetes/CPRD-Codelists/tree/pre-2024 (additional detail in Supplemental Material: Extended variable definitions).

### Treatment comparison

DPP4i and SU (pooled) were used as comparator drug classes as there is little evidence that they impact heart failure risk beyond their glucose-lowering effects (11–13) (the DPP4i saxagliptin may increase heart failure risk (14) but accounted for only 5.9% of the DPP4i group). We confirmed the validity of pooling both drug classes (see Sensitivity analyses).

Individuals were followed from treatment initiation until the earliest of: date of death, deregistration from primary care, last data collection from GP practice (maximum 15/10/2020), or five years post-drug initiation. We censored individuals if they initiated GLP1-receptor agonist or thiazolidinedione therapies. To ensure no overlap in follow-up time between treatment arms, individuals in the SGLT2i arm were censored if initiating DPP4i or SU, and individuals in the DPP4i/SU arm were censored if initiating SGLT2i. Individuals contributed follow-up time regardless of whether they adhered to treatment after initiation. Alternative censoring strategies were tested in sensitivity analyses.

### Outcome

The primary outcome was new-onset heart failure, defined as the earliest code for heart failure in primary care, hospital records or mortality records. This is a broader definition than the trial endpoint of hospitalisation for heart failure only, which we assessed in sensitivity analysis.

### Heart failure risk score

5-year absolute heart failure risk predictions were derived from QDiabetes-Heart Failure (QDiabetes-HF) 2015 (15). QDiabetes-HF was developed and validated in UK primary care data for the same primary outcome as above on over 500,000 patients with type 1 or 2 diabetes from 1998-2014, prior to widespread SGLT2i use. The model includes age, sex, ethnicity (White, Indian, Pakistani, Bangladeshi, Black Caribbean, Black African, Chinese, Other), deprivation, smoking status, diabetes type, diabetes duration, history of heart attack/angina/stroke, atrial fibrillation, chronic kidney disease stage 4/5, HbA1c, total cholesterol:HDL ratio, systolic blood pressure (SBP) and BMI as predictors. Cholesterol:HDL, SBP and BMI were imputed from age, sex, ethnicity, and smoking status where missing. All biomarker measurements were within the 2 years prior or up to 7 days after drug initiation.

### Weighting

To control for potential treatment selection bias, overlap weighting using propensity scores (16) and regression adjustment were used. The full covariate set used for both propensity scores and adjustment included: age, sex, ethnicity, deprivation score, smoking status, diabetes duration, atrial fibrillation, HbA1c, SBP, BMI, absolute heart failure risk (from QDiabetes-HF), hypertension, number of emergency inpatient hospital admissions in the previous year, number of ever and current glucose-lowering drug classes prescribed, current insulin use, and year of initiation. For regression adjustment, continuous covariates were modelled as non-linear 5-knot cubic splines. All analyses were complete case due to limited missingness (Supplemental Fig. 1).

### Statistical analysis

#### Evaluation of accuracy of SABRE model inputs

Calibration of QDiabetes-HF was evaluated across deciles of predicted heart failure risk by comparing to observed 5-year cumulative incidence of heart failure.

A meta-analysis of the 3 major SGLT2i trials in those with T2D and no ASCVD or heart failure (HF; >90% without history of HF (17–19)) reports a 37% lower risk of hospitalisation for heart failure hospitalisation (HR 0.63 [95%CI: 0.50–0.80]) (1). This meta-analysis did include up to 25% of patients (17; 20–23) with CKD and/or macroalbuminuria, unlike our study population. To assess consistency of the meta-analysis findings with our broader heart failure outcome, we used overlap-weighted adjusted Cox regression to estimate the overall hazard ratio comparing SGLT2 inhibitors with DPP4i/SU. We also explored whether the hazard ratio for heart failure varied by baseline absolute heart failure risk in a Cox model extended to incorporate a drug (SGLT2i versus DPP4i/SU) by QDiabetes-HF risk score (3-knot restricted cubic spline) interaction term. For all Cox models, proportional hazard assumptions were visually checked and confirmed.

#### Estimation and validation of SABRE-predicted absolute SGLT2i heart failure benefit

The SABRE model predicts 5-year absolute heart failure benefit with SGLT2i, derived by multiplying individual 5-year heart failure risk predictions from QDiabetes-HF with the relative SGLT2i risk reduction on heart failure from trial meta-analysis (37%). For example, for a patient with a 5-year QDiabetes-HF score of 20%, the SABRE model would predict an absolute benefit of 7.4% (20%*0.37).

To validate SABRE-predicted heart failure benefit, we fitted an overlap-weighted adjusted Cox model to the entire cohort, incorporating a term for treatment (SGLT2i or DPP4i/SU). We then used the model to estimate 5-year heart failure risk with both SGLT2i and DPP4i/SU for each individual, and derived observed benefits as the difference in predicted risk between SGLT2i and comparator. Calibration of median predicted and observed benefits was then assessed across deciles of predicted heart failure benefit.

#### Evaluation of treatment strategies based on the SABRE model versus current treatment guidelines

Expected heart failure outcomes with three binary (initiate SGLT2i, do not initiate SGLT2i) treatment strategies were evaluated:

1: International ADA/EASD guidance: treat individuals aged ≥55 years with 2 or more risk factors, including obesity, hypertension, or dyslipidaemia (2).
2: SABRE model aligned with ADA/EASD: treat individuals above a threshold of predicted heart failure benefit that results in recommending SGLT2i to the same proportion of individuals as ADA/EASD guidance.
3: SABRE model restricted strategy: treat individuals above a more restricted threshold than strategy 2, specifically the top 25% centile of predicted heart failure benefit in those recommended SGLT2i by ADA/EASD guidance.

We then calculated expected outcomes for each treatment strategy (alongside a ‘treat all’ strategy), comprising the proportion of the cohort who were SGLT2i treated, the absolute benefit in those treated (number of HF events prevented per 100 patient-years), and the number needed-to-treat per year to prevent one HF event (NNT). 95% confidence intervals for absolute benefits were estimated using bootstrap resampling (1000 iterations). For each strategy, we also estimated observed SGLT2i benefits as the difference in overlap-weighted 5-year cumulative incidence of heart failure with SGLT2i versus the comparator arm, fitted separately for those indicated and not indicated for treatment.

#### Sensitivity analyses

We performed the following sensitivity analyses of the relative heart failure benefit with SGLT2i to evaluate the robustness of our estimate:

1) To assess validity of the pooled comparator arm: SGLT2i versus SU-only and DPP4i-only comparators.
2) To assess using overlap weighting and multivariable adjustment to adjust for potential treatment bias: a) overlap weighting only without multivariable adjustment; b) multivariable adjustment alone; c) multivariable adjusted inverse probability weighting.
3) To assess the robustness of the overlap weighting and regression adjustment by additionally including i) microalbuminuria, and ii) estimated glomerular filtration rate, urine albumin:creatinine ratio and use of anti-hypertensive medications at baseline in weighting and adjustment covariate sets.
4) Using a more specific definition of hospitalisation or death with heart failure as the primary cause, which more closely resembles the outcome used in trials.
5) Including only individuals initiating second-line therapy after metformin (so individuals could only be in one of the SGLT2i, DPP4i or SU groups), with a) an intention-to-treat approach, and b) a per-protocol approach (censoring at earliest treatment change).
6) Allowing for death from non-heart failure causes as a competing risk.
7) Including only individuals co-treated with insulin.
8) Repeating the primary analysis in sex- and ethnicity-defined subgroups.

All analyses were conducted using R (v4.1.3; R Foundation for Statistical Computing, Austria). Reporting follows STROBE (24) and BePRECISE (25) guidelines which are provided in the Supplemental Material.

## Results

Amongst n=57,368 SGLT2i and n=111,673 DPP4i/SU comparator arm drug initiations (Supplemental Fig. 1) in those with type 2 diabetes (T2D) without atherosclerotic cardiovascular disease (ASCVD), heart failure (HF) or chronic kidney disease (CKD), median age was 58.0 years (IQR: 50.4–66.1 years), 57.6% were male, 74.6% were of White ethnicity, and median follow-up was 2.0 years (IQR: 0.9– 3.6 years). Overlap weighting resulted in well-balanced clinical features (Table 1, Supplemental Fig. 2).

### SGLT2i initiation is associated with a lower relative risk of new-onset heart failure, irrespective of baseline absolute heart failure risk

**Figure 1:**
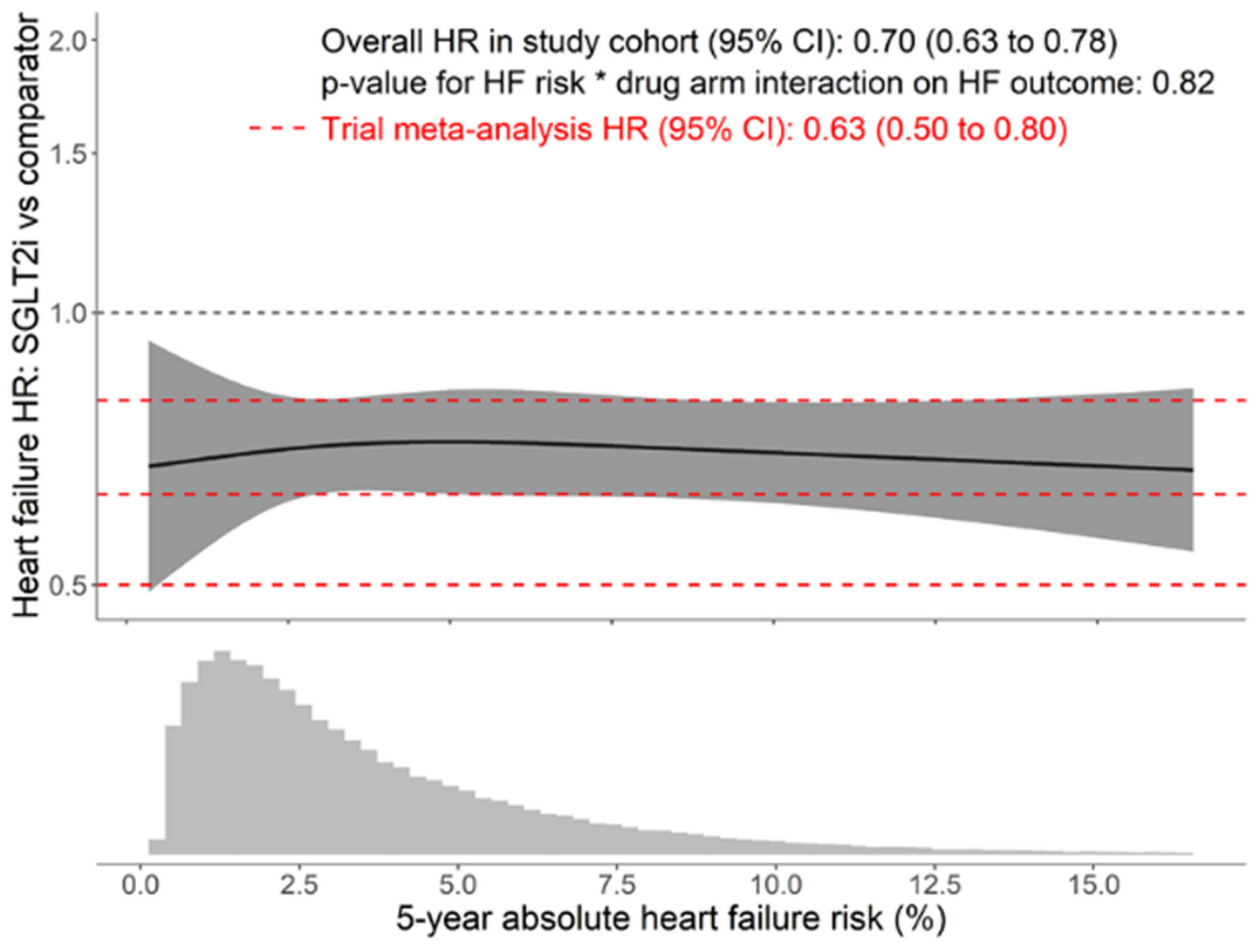
Hazard ratio estimates for new-onset heart failure for SGLT2-inhibitors vs comparator (DPP4-inhibitors/sulfonylurea) across baseline heart failure risk (model developed on n=169,041 [whole study cohort] using QDiabetes-Heart Failure for 5-year absolute heart failure risk estimates). Solid black line represents estimated hazard ratio (HR) at a given level of 5-year heart failure risk; grey shading represents 95% CI. Red dashed lines represent trial meta-analysis result with 95% CI. ‘Drug arm’ = SGLT2-inhibitor or comparator (DPP4-inhibitor/sulfonylurea). Lower plot shows distribution of baseline 5-year heart failure risk estimates within the study cohort.

1.2% (706/57,368) of SGLT2i initiators and 1.8% (2,052/111,673) of DPP4i/SU initiators experienced new-onset heart failure (incidence rates: SGLT2i: 5.60 per 1000 patient-years, DPP4i/SU: 7.78 per 1000 patient-years). Overall, risk of new-onset heart failure was 30% lower with SGLT2i versus DPP4i/SU (adjusted HR 0.70 [95% CI 0.63–0.78]), concordant with the 37% lower risk from previous trial meta-analysis. This lower risk was consistent across baseline heart failure risk (Fig. 1; p=0.82 for treatment arm:baseline HF risk interaction) and in all sensitivity analyses (HR range 0.61–0.73, Supplemental Fig. 3). Hazard ratios in non-White ethnicity subgroups had wider confidence intervals (Supplemental Table 2), due to small numbers.

### The QDiabetes-HF prediction model accurately predicts absolute risk of new-onset heart failure

5-year predictions of absolute heart failure risk were well-calibrated, although risk was slightly under-estimated in the highest risk decile (Supplemental Fig. 4). The C-statistic was 0.72 (95% CI 0.70–0.73) and the Brier score was 0.036 (95% CI 0.034– 0.038), supporting good calibration and accuracy.

### Absolute heart failure benefit predictions from the SABRE model are accurate in validation

**Figure 2:**
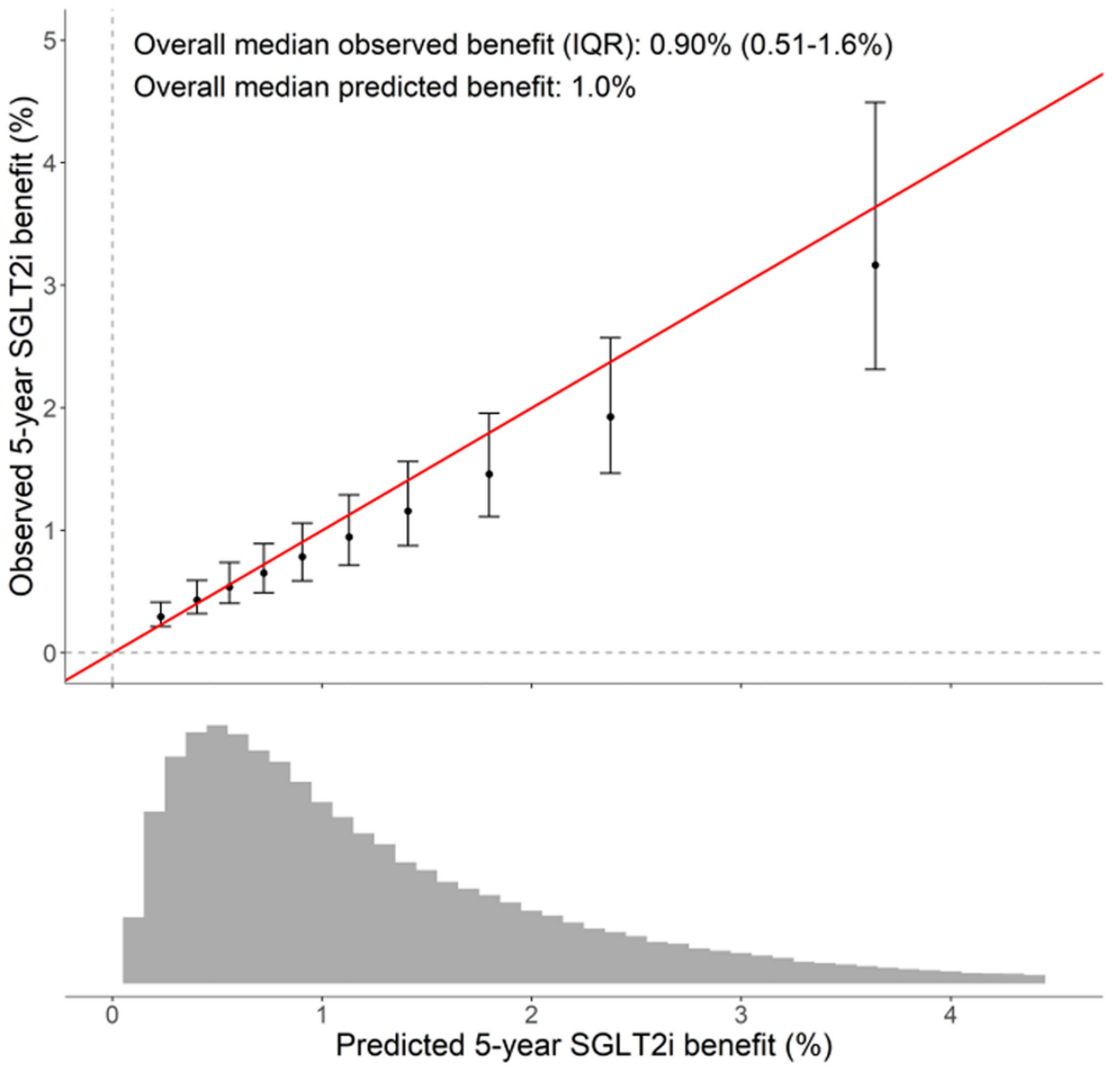
Calibration between 5-year predicted (from SABRE) and observed absolute SGLT2i benefit estimates in the study cohort. Values are medians by decile of predicted benefit, IQRs also shown for observed estimates (n=169,041). Lower plot shows distribution of 5-year predicted benefits (n=164,894; x-axis truncated at 4.5% excluding n=4,147 with predicted benefit >4.5%).

Predicted individual-level 5-year heart failure benefits ranged from <0.1% to 14.1% (median [IQR] 1.0% [0.6–1.8%]; Supplemental Fig. 5) and were well-calibrated with observed benefits (Fig. 2).

### SABRE allows more precise targeting of SGLT2i treatment for heart failure benefit compared with current guidelines

**Figure 3:**
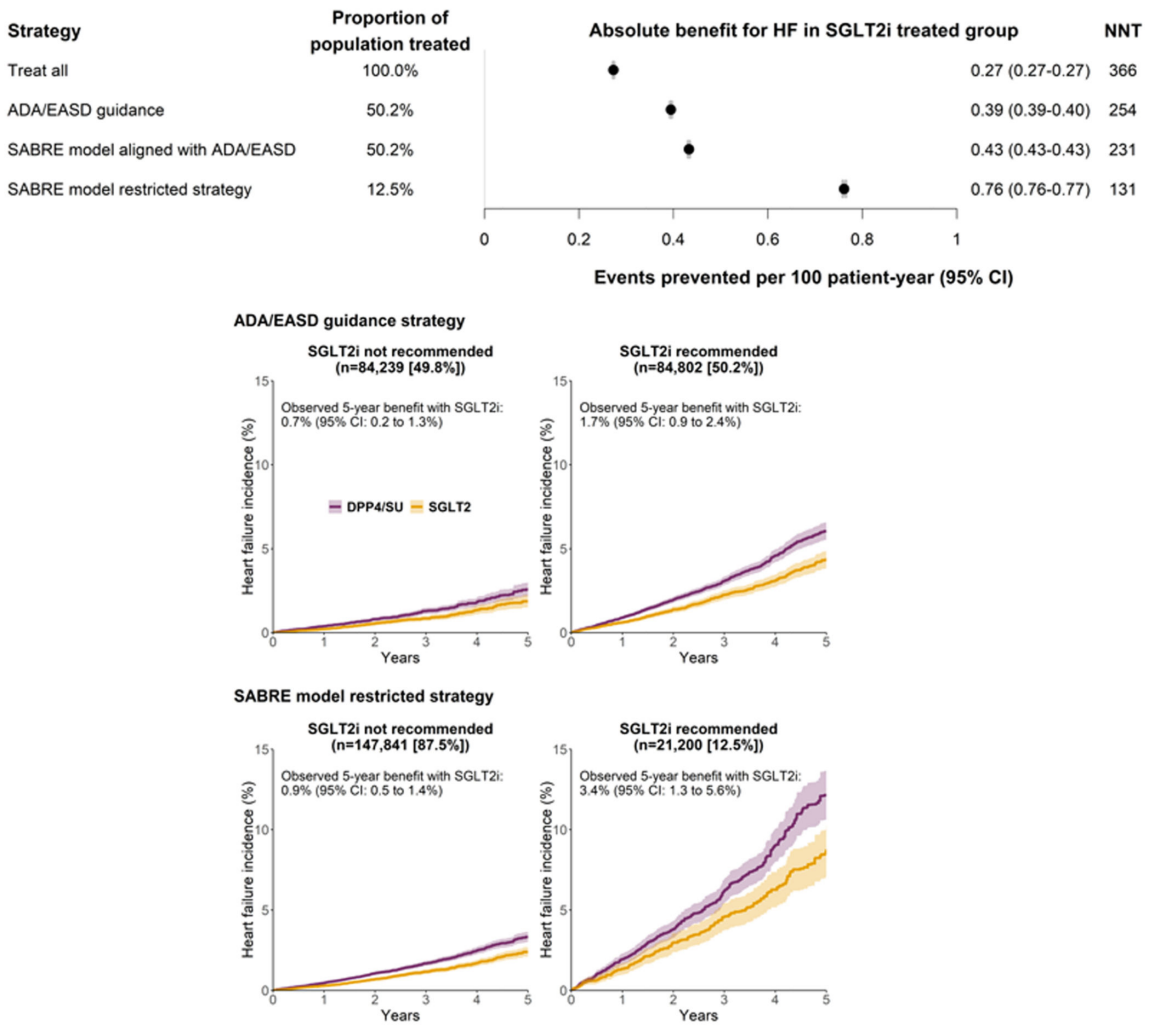
Evaluation of SGLT2i targeting strategies: A) Modelled absolute benefit (primary heart failure (HF) events prevented) in SGLT2i-treated individuals and B) Observed heart failure incidence over 5 years for SGLT2i-treated vs comparator (treated with DPP4-inhibitor/sulfonylurea). ADA-EASD guidance: treat individuals aged ≥55 years with 2 or more cardiovascular risk factors, including obesity, hypertension, or dyslipidaemia. SABRE model aligned with ADA/EASD: treat individuals with SABRE predicted heart failure benefit >1.0%, resulting in recommending SGLT2i to the same proportion of individuals as ADA/EASD guidelines. SABRE model restricted strategy: Treat individuals with SABRE predicted heart failure benefit >2.6%, corresponding to the top 25% centile of predicted heart failure benefit in those recommended SGLT2i by ADA/EASD guidelines. A) Absolute benefit is number of primary HF events prevented per 100 patient-years of treatment in those treated, derived from SABRE model predictions; NNT is number of patients needed-to-treat per year to prevent one HF event. B) Overlap weighting used within each stratum to balance SGLT2i and comparator groups; observed benefits are estimated differences in survival at 5 years between the weighted SGLT2i and comparator groups.

The expected outcomes of different treatment strategies, in terms of the proportion of those with T2D without ASCVD/HF/CKD who would be SGLT2i-treated, absolute benefit for those treated, and numbers needed-to-treat, are shown in Fig. 3A. The results show that deploying the SABRE model at a threshold aligned with current ADA/EASD guidance (treat 50% of patients; predicted 5-year heart failure benefit >0.48%) results in a lower 1-year NNT (231 vs 254 for ADA/EASD), with an increase in HF events prevented from 0.39 [95% CI 0.39–0.40] to 0.43 [95% CI 0.43–0.43] events per 100 patient-years in those treated.

In addition, the SABRE model allows development of more targeted treatment strategies, further lowering the NNT in those treated. This is illustrated by the SABRE model restricted strategy, where targeting the top quartile of predicted benefit in those indicated by ADA/EASD guidance reduces 1-year NNT to 131, with 0.65 (95% CI 0.65–0.66) HF events prevented per 100 patient-years in the SGLT2i-treated.

Fig. 3B shows observed 5-year incidence of heart failure with treatment initiation defined by ADA/EASD guidance and the SABRE restricted strategy (see Supplemental Fig. 6 for SABRE model aligned with ADA/EASD strategy). Observed heart failure benefits with SGLT2i versus DPP4i/SU were greater in those receiving treatment with the SABRE restricted strategy (3.4% benefit, 95% CI 1.3–5.6%) than the ADA/EASD guidance strategy (1.7% benefit, 95% CI 0.9–2.4%). Despite the higher proportion of individuals not indicated for treatment under the SABRE strategy, observed outcomes in those not recommended for treatment were similar (SABRE: 0.9% lower absolute risk in SGLT2i arm, ADA/EASD: 0.7%).

## Conclusions

### Principal findings

We develop and validate a SGLT2i heart failure benefit prediction model (SABRE) allowing accurate identification of individuals who would benefit most from SGLT2i for primary heart failure prevention. The model is applicable to people with type 2 diabetes without atherosclerotic cardiovascular disease (ASCVD), heart failure (HF) or chronic kidney disease (CKD), which represent the majority of people with T2D. Importantly, we establish that although there is no evidence of variation in the relative SGLT2i benefit for heart failure in our large population-representative T2D population without ASCVD/HF/CKD, there is substantial variation in absolute benefit, and this allows for prioritisation of SGLT2i to those with greatest predicted absolute benefit. In our UK cohort, SABRE enabled more precise targeting than existing SGLT2i targeting strategies from ADA/EASD (2) and NICE (3). Direct low-cost implementation of SABRE is possible as the model combines RCT results with an established risk prediction model based on routine clinical features which can easily be updated for deployment in other settings.

### Strengths and weaknesses compared to existing studies

Our observational finding of an overall 30% lower risk of new-onset heart failure in those with T2D and no pre-existing ASCVD with SGLT2i (HR 0.70 [95% CI 0.63– 0.78]) is consistent with previous RCT meta-analysis (1; 26) (HR 0.63 [95% CI 0.49– 0.80] for first hospitalisation for heart failure in both meta-analyses). Importantly, we show this average estimate appears constant across the range of continuous baseline heart failure risk seen in our large multi-ethnic and population-representative T2D population. This consistent relative benefit has been previously suggested across subgroups defined by baseline heart failure risk in RCT data (27) and supports potential generalisability of the SABRE model to other populations in which heart failure risk patterns may be different to the UK. A recent study has suggested that whilst ASCVD is decreasing in those with T2D, heart failure incidence has plateaued or even started to increase, especially in younger patients (28), supporting heart failure as a relevant treatment target.

SABRE predicted that treating our entire study population with SGLT2i would prevent 0.27 (95% CI 0.27–0.27) HF events per 100 patient-years; less than the 0.39 (95% CI 0.32–0.47) events per 100 patient-years reported in an existing meta-analysis of observational studies in those with T2D and no ASCVD (29), likely reflecting lower baseline heart failure risk in our study population. The same meta-analysis showed a much greater benefit in those with T2D and ASCVD (for whom SGLT2i are currently recommended) of 1.17 (95% CI 0.78–1.55) HF events prevented per 100 patient-years (29). In our study, targeting SGLT2i to the 12.5% of those with T2D without ASCVD/HF/CKD with greatest predicted benefit increased the benefit to 0.76 (95% CI 0.76–0.77) HF events prevented per 100 patient-years, still below the benefit seen in those with T2D and ASCVD.

### Strengths and weaknesses

Strengths of this study include the use of existing evidence on SGLT2i benefit from clinical trial meta-analysis and a validated risk prediction model to develop the SABRE model. The final model was then validated in a large, population-representative dataset. We focused on individuals with T2D who do not have ASCVD, HF, or CKD, as evidence-based guidance already recommends initiation of SGLT2i for individuals with these conditions. In contrast to the original RCTs included in the trial meta-analysis, in which up to 25% of patients had CKD and/or macroalbuminuria, our study population excluded those with chronic kidney disease (stage 3a–5) or macroalbuminuria as they are already indicated for SGLT2i under current guidance. Our finding of a similar relative risk reduction with SGLT2i for HF as the meta-analysis therefore suggests that the presence of CKD and/or macroalbuminuria does not substantially affect the relative benefit of SGLT2i for heart failure.

A limitation of our study is that both the study population used here to validate SABRE, and those used in the development of QDiabetes-HF, were of majority White ethnicity and aged ≤84 years. There is limited evidence from RCTs on the relative effectiveness of SGLT2i for cardiovascular outcomes across different ethnicities (30) and particular for primary heart failure prevention in T2D. Although we found that SGLT2i was associated with greater reductions in heart failure across all non-White ethnicity groups compared to White ethnicity in our study (ESM Table 2), patient numbers in non-White groups were limited, and the need for ethnicity-specific models should be carefully evaluated in larger datasets in future work. In addition, the performance of the QDiabetes-HF model used in SABRE to predict heart failure risk was modestly lower (C-statistic: 0.72) in our study population than in the original validation (0.77–0.78 (6)), which may reflect temporal changes since model development and/or differences between the development cohort and our study cohort. Routinely available risk factors for heart failure including chronic obstructive pulmonary disease, obstructive sleep apnoea, and excessive alcohol use (31) are not included in QDiabetes-HF and could improve accuracy of predictions.

We also did not evaluate the potential of NT-proBNP, a well-established biomarker for heart failure risk and prognosis (32). Future work could examine whether incorporating NT-proBNP improves the predictive performance of SABRE, and how NT-proBNP performs alone compared to SABRE. However, the lack of routine NT-proBNP testing in the UK currently limits the feasibility of this analysis with our study dataset. Where available, SABRE could be updated with new features or even underlying heart failure risk prediction models to maximise clinical utility.

Another limitation of our study is that validation was performed using observational data of patients receiving routine care (i.e. not randomised to treatment). This introduces the possibility of residual confounding and treatment bias which will not have been fully accounted for in our analysis. Ideally, the findings would be validated in a randomised controlled trial; however, the limited number of participants in existing trials with T2D but without established ASCVD, HF, or CKD restricts the feasibility of such validation in currently available data. Despite this, the concordance between previous RCT results and our estimates of SGLT2i-associated heart failure benefit suggests that any remaining bias is unlikely to explain our results. We have not specifically examined differences in SGLT2i dose or adherence, which may impact heart failure outcomes. However, our data reflects real-world clinical practice and patient behaviours and so represents typical UK prescribing patterns and medication use.

### Potential implications for clinicians or policymakers

The SABRE model demonstrates a novel approach based on individual-level risk estimation to support targeting of SGLT2i within the majority of the T2D population, who do not have ASCVD, HF or CKD, to those who would benefit the most for heart failure prevention. This group of patients represent a significant and increasing population for whom current preventative guidance is largely based on ASCVD outcomes rather than heart failure, and is inconsistent across the US, UK and Europe (2; 3). Although our study focused on heart failure, an important point is that those at highest risk of heart failure are generally also at highest risk of other CVD outcomes (33), and so prioritising treatment based on heart failure risk will largely align with a broader CVD-targeted strategy.

Although we developed and validated SABRE in UK data, simple deployment is possible in other populations. Before deployment, accuracy assessment using setting-specific data, or replacement of the underlying absolute risk model with one that has been locally validated, will be required. An advantage of the model is that the number of patients indicated for treatment can be flexibly adjusted by changing the benefit threshold used, to align with local or national cost considerations. Of note, even in those with substantial predicted heart failure benefit with SGLT2i, individual treatment decisions should carefully consider the potential side effects of SGLT2i, especially in frail or elderly individuals (34), as well as other benefits including glycaemic response (35) and weight improvement.

### Unanswered questions and future research

In addition to heart failure benefits, SGLT2i have demonstrated significant benefits for kidney protection (1). Whilst our current study focused specifically on heart failure, a recent analysis has shown similar potential for using risk scores to predict individual-level kidney protection benefit with SGLT2i (36). Despite heart failure and chronic kidney disease sharing many risk factors (37), there are likely to be patients who would benefit substantially from SGLT2i for kidney protection but are not identified by the current SABRE model as having high predicted heart failure benefit. Extending SABRE to incorporate a broad range of clinically-relevant (e.g. kidney and cardiovascular) outcomes could provide important new insights into patient stratification and inform development of more individualised clinical guidelines and practice.

Our analysis did not incorporate GLP-1 receptor agonists (GLP-1RA), for which next generation agents have recently been shown to reduce the risk of atherosclerotic cardiovascular disease (ASCVD) and heart failure (the latter to a lesser degree than SGLT2i) (38). In ADA/EASD guidance, GLP-1RA are recommended as an alternative to SGLT2i for those with T2D at high CVD risk (2), whereas in the UK they are currently only recommended for limited subset of those with T2D (predominantly those with obesity) (3). As both drug classes have also been shown to reduce heart failure and cardiovascular disease in individuals without diabetes (38; 39), future studies applying our research framework to understand the absolute benefits in both diabetes and non-diabetes populations would be of considerable interest.

Finally, further validation of the SABRE model in non-White and older populations will be important to ensure generalisability and equity, particularly given known ethnic and age-related differences in heart failure risk, treatment response, and access to care.

### Conclusion

We have developed and validated the SABRE model to predict the absolute benefit for heart failure prevention of initiating SGLT2i for people with type 2 diabetes without ASCVD, heart failure or chronic kidney disease. The model framework, based on RCT evidence and validated clinical prediction models, means it can be quickly adapted for deployment in multiple countries. Deployment could support a more targeted approach to prescribing of SGLT2i in people with type 2 diabetes to maximise population-level benefit.

## Supplementary Material

Supplemental material

## Figures and Tables

**Table 1:** Baseline characteristics of study cohort at drug (SGLT2-inhibitor/comparator) initiation after overlap weighting. Unweighted characteristics shown in Supplemental Table 1. All values are % or median (interquartile range).

	Comparator (DPP4i/SU)	SGLT2i
**Sex (% male)**	57.2%	57.2%
**Age at drug initiation (years)**	57.1 (49.6-65.2)	57.3 (50.2-64.6)
**Diabetes duration (years)**	6.8 (3.6-11.1)	6.9 (3.8-11.1)
**Ethnicity**		
White	75.4%	75.4%
Asian	14.1%	14.1%
Black	4.5%	4.5%
Mixed/Other	3.6%	3.6%
Missing	2.5%	2.5%
**Index of Multiple Deprivation quintile**		
1 (least deprived)	16.9%	16.9%
2	17.7%	17.7%
3	19.2%	19.2%
4	22.5%	22.5%
5 (most deprived)	23.7%	23.7%
**Smoking status**		
Non-smoker	52.8%	52.8%
Active smoker	16.2%	16.2%
Ex-smoker	31.1%	31.1%
**Hypertension**	53.9%	53.9%
**Atrial fibrillation**	2.3%	2.3%
**Number of hospital admissions in previous year**		
0	80.9%	80.9%
1	16.5%	16.5%
2+	2.6%	2.6%
**BMI (kg/m^2^)**	32.1 (28.2-37.0)	32.4 (28.9-36.9)
**HbA1c (%)**	8.7 (7.9-10.0)	8.8 (8.0-10.0)
**HbA1c (mmol/mol)**	72.0 (63.0-86.0)	73.0 (64.0-86.0)
**SBP (mmHg)**	132.0 (124.0140.0)	132.0 (124.0140.0)
**Total cholesterol:HDL**	3.8 (3.1-4.7)	3.8 (3.1-4.7)
Missing	3.6%	3.5%
**Drug line**		
2	36.8%	36.8%
3	38.8%	38.8%
4	17.8%	17.8%
5+	6.7%	6.7%
**Number of other current non-insulin glucose-lowering medications**		
0	9.0%	9.0%
1	57.2%	57.2%
2+	33.8%	33.8%
**Current insulin use**	5.3%	5.3%
**Year of drug initiation**		
2013	2.6%	2.6%
2014	8.0%	8.0%
2015	12.9%	12.9%
2016	14.3%	14.3%
2017	16.1%	16.1%
2018	17.6%	17.6%
2019	18.1%	18.1%
2020	10.3%	10.3%
**QDiabetes-Heart Failure 5-year score (%)**	2.7 (1.5-4.8)	2.7 (1.5-4.7)

## Data Availability

The data that support the findings of this study are available from CPRD, subject to protocol approval via CPRD’s research data governance process (https://cprd.com/data-access). Code for initial cohort preparation is available at: https://github.com/Exeter-Diabetes/CPRD-Cohort-scripts/tree/Oct2020-download/03-Treatment-response-(MASTERMIND) and analysis code is available at https://github.com/Exeter-Diabetes/CPRD-Katie-SGLT2-HF. The QDiabetes-Heart Failure algorithm is available as open-source software under the GNU Aferro General Public Licence, version 3 from https://qdiabetes.org/heart-failure/index.php.

## Twitter summary

The SABRE model combines QDiabetes-HF and trial evidence to predict absolute SGLT2i heart failure benefit in type 2 diabetes. Targeting SGLT2i with SABRE is more efficient than current guidelines.

## Article highlights

### Why did we undertake this study?

- Most people with type 2 diabetes (T2D) have no cardiovascular disease, heart failure, or kidney disease (ASCVD/HF/CVD). Current SGLT2-inhibitor guidelines for this group are broad and do not identify which individuals are most likely to benefit.

### What is the specific question(s) we wanted to answer?

- Can we accurately predict which individuals with T2D without ASCVD/HF/CKD would gain the greatest absolute reduction in heart-failure risk from SGLT2-inhibitors?

### What did we find?

- We developed and validated the SGLT2-inhibitor absolute benefit response (SABRE) model, which estimates 5-year absolute heart-failure benefit using routine risk factors and RCT-derived treatment effects.

### What are the implications of our findings?

- SABRE provides precise, individualised targeting of SGLT2-inhibitors for primary prevention of heart-failure in people with T2D.
