# Supplementary material for: Precision prescribing of SGLT2-inhibitors in people with type 2 diabetes for primary prevention of heart failure: model development and validation study
Source: Diabetes Care. Author manuscript; Available in PMC 2026 Jun 13. (PMC7619156; doi:10.2337/dc25-2973)
Supplement: Supplemental material [file EMS214145-supplement-Supplemental_material.pdf]

SUPPLEMENTAL MATERIAL

Precision prescribing of SGLT2-inhibitors in people with type 2 diabetes for primary prevention of heart failure: model development and validation study

Katherine G Young[1], Andrew P McGovern[1], Rhian Hopkins[1], Thijs T Jansz[1], Pedro M Cardoso[1], Rury R Holman[2], Ewan R Pearson[3], Andrew T Hattersley[1], Angus G Jones[1], Kieran Docherty[4], Naveed Sattar[4], Beverley M Shields[1], John M Dennis[1], on behalf of the MASTERMIND Consortium

[1]Clinical and Biomedical Sciences, University of Exeter Medical School, Exeter, UK
[2]Diabetes Trials Unit, Radcliffe Department of Medicine, University of Oxford, Oxford, UK
[3]Division of Diabetes, Endocrinology and Reproductive Medicine, Ninewells Hospital and Medical School, University of Dundee, Dundee, UK
[4]School of Cardiovascular and Metabolic Health, University of Glasgow, Glasgow, UK

Extended variable definitions

All codelists for defining baseline variables used for QDiabetes-Heart Failure, regression model adjustment/propensity scores, as well as heart failure outcomes, can be found here: https://github.com/Exeter-Diabetes/CPRD-Codelists/tree/pre-2024. In addition, this repository has algorithms for defining date of birth, diabetes type, diabetes duration, ethnicity, smoking status, chronic kidney disease status, as well as cleaning rules for diagnoses, prescriptions and biomarkers.

Additional information on variables not fully defined by the above:

- Cardiovascular disease was present at baseline if there was any record of angina, ischaemic heart disease, myocardial infarction, peripheral arterial disease, revascularisation, stroke or transient ischaemic attack in primary or secondary care data prior to baseline.
- Heart failure was present at baseline if there was any record of heart failure in primary or secondary care data prior to baseline.
- Chronic kidney disease was present at baseline if there was any record of chronic kidney disease stage 3a-5 or end stage renal disease or macroalbuminuria (urinary albumin:creatinine >30 mg/mmol) in primary or secondary care data prior to baseline.
- Ethnicity was coded as 9-category QDiabetes-HF ethnicity (NB: the model treats missing ethnicity as White).
- Smoking status was coded as 5-category QDiabetes-HF smoking status.
- For defining current medication use, gaps of <6 months between subsequent prescriptions for the same drug class were ignored (represented continuous treatment) as per our previously published protocol (1).
- Drug initiations within 3 months of patient registration at the primary care practice were excluded as they may not represent new initiations.
- Number of glucose-lowering drug classes ever prescribed was categorised as 1, 2, 3, 4 or 5+ distinct glucose-lowering drug classes (including the current drug being initiated).
- Number of other current non-insulin glucose-lowering medications (metformin, sulfonylurea, DPP4-inhibitor, SGLT2-inhibitors, thiazolidinedione or GLP-1 receptor agonist; not including drug [SGLT2i/DPP4i/SU] being initiated) was categorised as 0, 1 or 2+.
- Number of emergency inpatient hospital admissions in the previous year (admissions recorded in Hospital Episode Statistics Admitted Patient Care [HES APC] data with an admission method other than ‘elective’) was categorised as 0, 1-2 or 3+.
- Deprivation scores: 2015 English Index of Multiple Deprivation (IMD) decile scores were used for propensity scores and hazard ratio adjustment. Townsend Deprivation Scores (TDS) were required for QDiabetes-HF. To estimate TDS scores from IMD, we used 2015 IMD deciles and 2011 TDS scores for all Lower Super Output Areas (LSOAs) in England, and found the median 2011 TDS corresponding to each IMD decile score.
- Microalbuminuria (used for ADA-EASD high cardiorenal risk definition and weighting/adjustment in sensitivity analysis) was defined as urinary

albumin:creatinine ratio of ≥3 mg/mmol and ≤30 mg/mmol (assumed to be normal if missing).

- Current use of blood pressure medication was defined as prescription for an angiotensin-converting enzyme inhibitor, angiotensin II receptor blocker, loop diuretic, potassium-sparing diuretic, or thiazide-like diuretic in the year prior to baseline
- Obesity (used for ADA-EASD high cardiorenal risk definition) was defined as BMI >30 kg/m^2.
- Hypertension (used for adjustment/weighting and ADA-EASD high cardiorenal risk definition) was defined as a previous diagnosis of hypertension in primary care data or SBP>140 mmHg or DBP>90 mmHg (SBP and DBP assumed to be normal if missing).
- Dyslipidaemia (used for ADA-EASD high cardiorenal risk definition) was defined as current statin treatment (as above, gaps of <6 months between subsequent prescriptions were ignored) or total cholesterol >5 mmol/L.
- Total cholesterol:HDL ratio was calculated from separate total cholesterol and HDL codes and imputed from age, sex, ethnicity and smoking status where missing as per the QDiabetes-Heart Failure algorithm.
- Patients with biomarker values outside the range allowed by QDiabetes-HF were excluded (HbA1c: <5.8 or >15.9% (<40 or >150 mmol/mol), cholesterol:HDL <1 or >11 mmol/L, SBP <70 or >210 mmHg).
- All biomarkers (HbA1c, total cholesterol, cholesterol:HDL, SBP, DBP, BMI, urinary albumin:creatinine ratio) were within the 2 years prior or up to 7 days after baseline date. HbA1c measurements were taken a median of 15 days before drug initiation, BMI 27 days, total cholesterol 32 days, HDL 35 days, SBP 13 days, DBP 13 days, urinary albumin:creatinine ratio 123 days; 83.6% of biomarker measurements were within 6 months of drug initiation.

Supplemental Tables

Supplemental Table 1: Baseline characteristics of study cohort at drug (SGLT2-inhibitor/comparator) initiation (no weighting).

	Comparator (DPP4i/SU) (N=111,673; N=68,708 [61.5%] DPP4i, N=42,965 [38.5%] SU)	SGLT2i (N= 57,368)
**Sex (% male)**	64,473 (57.7%)	33,041 (57.6%)
**Age at drug initiation (years)**	58.6 (50.6-67.0)	57.1 (50.2-64.2)
**Diabetes duration (years)**	6.1 (3.1-10.0)	8.2 (4.6-12.5)
**Ethnicity**		
White	82,817 (74.2%)	43,346 (75.6%)
Asian	16,129 (14.4%)	8,194 (14.3%)
Black	5,987 (5.4%)	2,419 (4.2%)
Mixed/Other	4,214 (3.8%)	1,967 (3.4%)
Missing	2,526 (2.3%)	1,442 (2.5%)
**Index of Multiple Deprivation quintile**		
1 (least deprived)	18,200 (16.3%)	9,961 (17.4%)
2	19,744 (17.7%)	10,191 (17.8%)
3	21,136 (18.9%)	11,016 (19.2%)
4	25,491 (22.8%)	12,840 (22.4%)
5 (most deprived)	27,102 (24.3%)	13,360 (23.3%)
**Smoking status**		
Non-smoker	58,224 (52.1%)	30,599 (53.3%)
Active smoker	18,836 (16.9%)	8,905 (15.5%)
Ex-smoker	34,613 (31.0%)	17,864 (31.1%)
**Hypertension**	59,321 (53.1%)	31,526 (55.0%)
**Atrial fibrillation**	2,945 (2.6%)	1,281 (2.2%)
**Number of hospital admissions in previous year**		
0	88,697 (79.4%)	46,871 (81.7%)
1	19,301 (17.3%)	9,158 (16.0%)
2+	3,675 (3.3%)	1,339 (2.3%)
**BMI (kg/m2)**	31.0 (27.4-35.5)	32.7 (29.0-37.2)
**HbA1c (%)**	8.6 (7.8-9.9)	8.9 (8.1-10.1)
**HbA1c (mmol/mol)**	71.0 (62.0-85.0)	74.0 (65.0-87.0)
**SBP (mmHg)**	132.0 (123.0-140.0)	132.0 (124.0-140.0)
**Total cholesterol:HDL**	3.8 (3.1-4.7)	3.8 (3.1-4.6)
Missing	4604 (4.1%)	2015 (3.5%)
**Drug line**		
2	62,881 (56.3%)	13,474 (23.5%)
3	37,750 (33.8%)	17,439 (30.4%)
4	8,675 (7.8%)	15,996 (27.9%)
5+	2,367 (2.1%)	10,459 (18.2%)
**Number of other current non-insulin glucose-lowering medications**		

0	12,453 (11.2%)	4,235 (7.4%)
1	73,741 (66.0%)	27,181 (47.4%)
2+	25,479 (22.8%)	25,952 (45.2%)
**Current insulin use**	2,685 (2.4%)	6,051 (10.5%)
**Year of drug initiation**		
2013	16,028 (14.4%)	862 (1.5%)
2014	15,233 (13.6%)	3,870 (6.7%)
2015	16,069 (14.4%)	7,124 (12.4%)
2016	15,943 (14.3%)	7,822 (13.6%)
2017	15,351 (13.7%)	8,974 (15.6%)
2018	14,423 (12.9%)	10,411 (18.1%)
2019	12,092 (10.8%)	11,688 (20.4%)
2020	6,534 (5.9%)	6,617 (11.5%)
**QDiabetes-Heart Failure 5-year score (%)**	2.7 (1.5-5.0)	2.8 (1.6-4.8)

All values are n (%) or median (interquartile range).

DPP4i=DPP4-inhibitor, SGLT2i=SGLT2i-inhibitor, SU=sulfonylurea.

Supplemental Table 2: Events counts and hazard ratio estimates for new-onset heart failure for SGLT2-inhibitors vs comparator (DPP4-inhibitors/sulfonylurea) in the study cohort compared to trial meta-analysis, stratified by ethnicity subgroup. All models are adjusted with overlap weighting using propensity scores unless otherwise stated (see 'Weighting' section of Methods for variables used for adjustment and propensity scores).

Self-reported ethnicity	SGLT2i			Comparator (DPP4i/SU)			Hazard ratio (95% CI)
	N	HF events	Incidence rate [1]	N	HF events	Incidence rate [1]	
White	43,346	609	6.34	82,817	1,706	8.79	0.71 (0.63 to 0.80)
Asian	8,194	68	3.81	16,129	200	5.11	0.64 (0.45 to 0.92)
Black	2,419	18	3.56	5,987	75	5.14	0.62 (0.34 to 1.14)
Mixed/ Other	1,967	8	1.98	4,214	61	6.21	0.28 (0.11 to 0.72)

DPP4i: DPP4-inhibitor, HF: heart failure, SGLT2i: SGLT2-inhibitor, SU: sulfonylurea.

[1] per 1000 patient-years

Supplemental Figures

Supplemental Figure 1: Flow diagram of inclusion for the study cohort

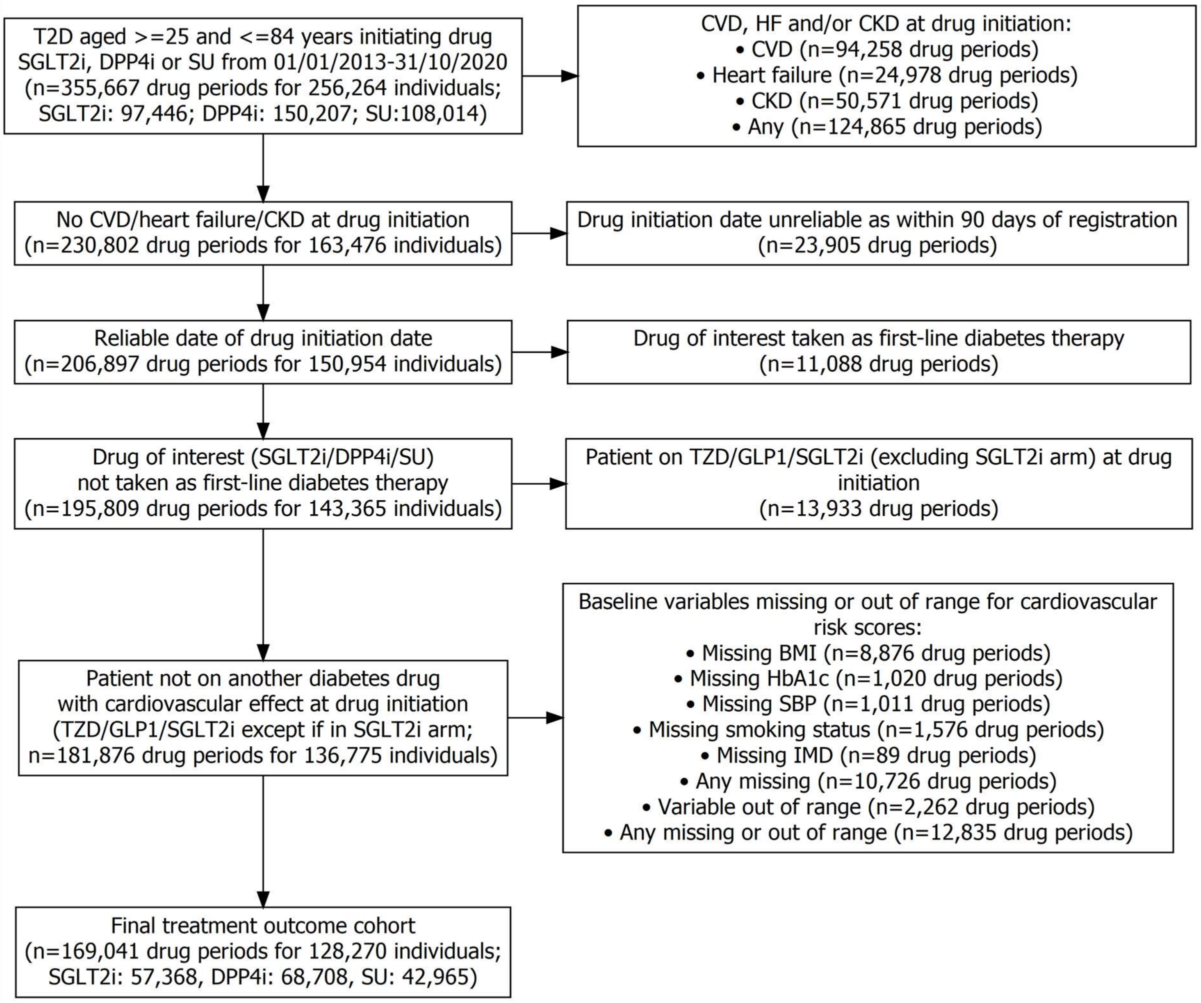

CKD=chronic kidney disease, CVD=cardiovascular disease, DPP4i=DPP4-inhibitor, GLP1=GLP1 receptor agonist, HF=heart failure, SGLT2i=SGLT2i-inhibitor, SU=sulfonylurea, T2D=type 2 diabetes, TZD=thiazolidinedione.

Supplemental Figure 2: Love plot of covariate balance in the SGLT2-inhibitor and comparator (DPP4-inhibitor/sulfonylurea; reference group) groups of the treatment outcome cohort before and after overlap weighting.

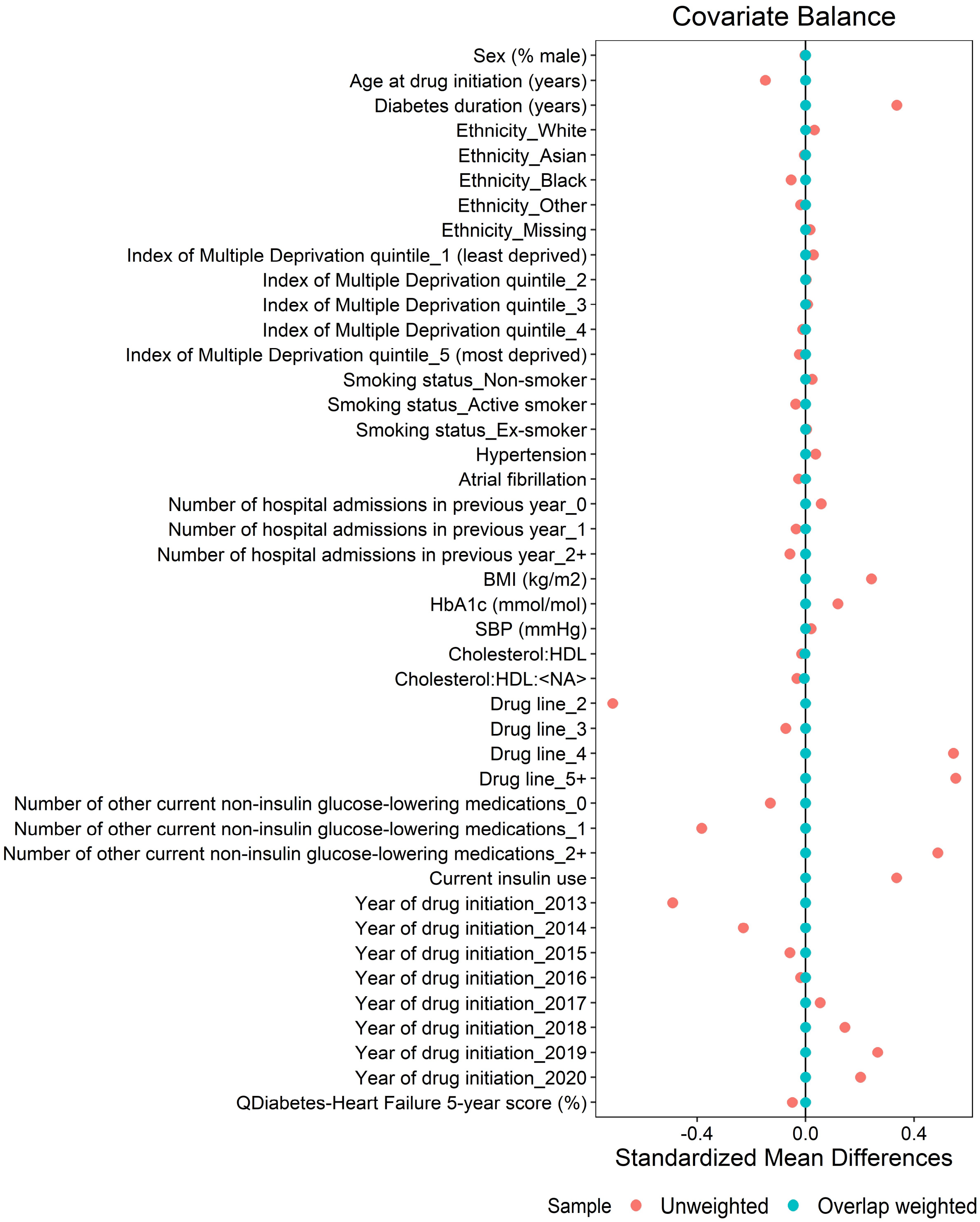

Supplemental Figure 3: Hazard ratio estimates for new-onset heart failure for SGLT2-inhibitors vs comparator (DPP4-inhibitors/sulfonylurea) in the study cohort compared to trial meta-analysis. All models are adjusted with overlap weighting using propensity scores unless otherwise stated (see 'Weighting' section of Methods for variables used for adjustment and propensity scores).

	SGLT2i Events	IR	Comparator Events	IR	Hazard ratio (95% CI)
Primary analysis: adjusted with overlap weighting	706/57368	5.60	2052/111673	7.78	0.70 (0.63-0.78)
SU-only comparator group	706/57368	5.60	826/42965	7.69	0.69 (0.60-0.80)
DPP4i-only comparator group	706/57368	5.60	1226/68708	7.84	0.70 (0.62-0.79)
Unadjusted with overlap weighting	706/57368	5.60	2052/111673	7.78	0.68 (0.61-0.76)
Adjusted with no weighting	706/57368	5.60	2052/111673	7.78	0.70 (0.63-0.78)
Adjusted with IPTW	706/57368	5.60	2052/111673	7.78	0.67 (0.59-0.76)
Additional adjustment for microalbuminuria	706/57368	5.60	2052/111673	7.78	0.70 (0.63-0.78)
Additional adjustment for eGFR, UACR, BP medication[1]	592/47209	5.67	1704/92894	7.64	0.73 (0.65-0.82)
More specific HF definition[2]	147/57368	1.16	440/111673	1.65	0.65 (0.51-0.84)
Second-line intention-to-treat[3]	105/11537	3.97	1155/52767	6.54	0.73 (0.59-0.90)
Second-line per-protocol[3]	44/11537	2.88	470/52767	5.32	0.61 (0.43-0.84)
Death as a competing risk[4]	706/57368	5.60	2052/111673	7.78	0.71 (0.64-0.79)
Insulin-treated subgroup only	151/6051	9.57	99/2685	14.95	0.67 (0.49-0.93)
Males only	429/33041	5.82	1207/64473	7.77	0.68 (0.59-0.79)
Females only	277/24327	5.28	845/47200	7.79	0.72 (0.60-0.86)
Trial meta-analysis					0.63 (0.50-0.80)

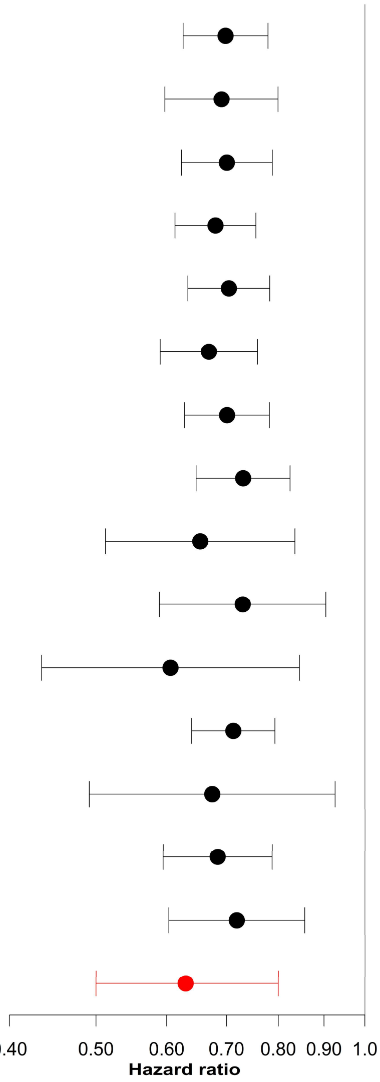

BP medication: blood pressure (anti-hypertensive) medication, DPP4i: DPP4-inhibitor, eGFR: estimated glomerular filtration rate, HF: heart failure, IPTW: inverse probability of treatment weighting, IR: incidence rate per 1000 patient years, SGLT2i: SGLT2-inhibitor, SU: sulfonylurea, UACR: urine albumin:creatinine ratio.

[1]Patients without measured eGFR (n=428) or UACR (n=28,778) at baseline excluded.

[2]Outcome includes death or hospitalisation with HF as the primary cause only (main analyses: any code for HF in primary or secondary care or death causes included).

[3]Patients on SGLT2i/DPP4i/SU second-line after metformin. Intention-to-treat: not censored at glucose-lowering treatment change; per-protocol: censored at earliest glucose-lowering treatment change (main analysis: censored if initiate one of the other study drugs, GLP1-receptor agonist or thiazolidinedione).

[4]Death from non-heart failure causes: SGLT2i IR: 5.55, Comparator IR: 9.72. Subdistribution hazard ratio reported as hazard ratio.

Supplemental Figure 4: Predicted 5-year absolute risk of new-onset heart failure (from QDiabetes-Heart Failure; median per decile) vs observed estimates for DPP4-inhibitor/sulfonylurea treatment arm (n=111,673). C-statistic: 0.72 (95% CI: 0.70 to 0.73); Brier score: 0.036 (95% CI: 0.034 to 0.038).

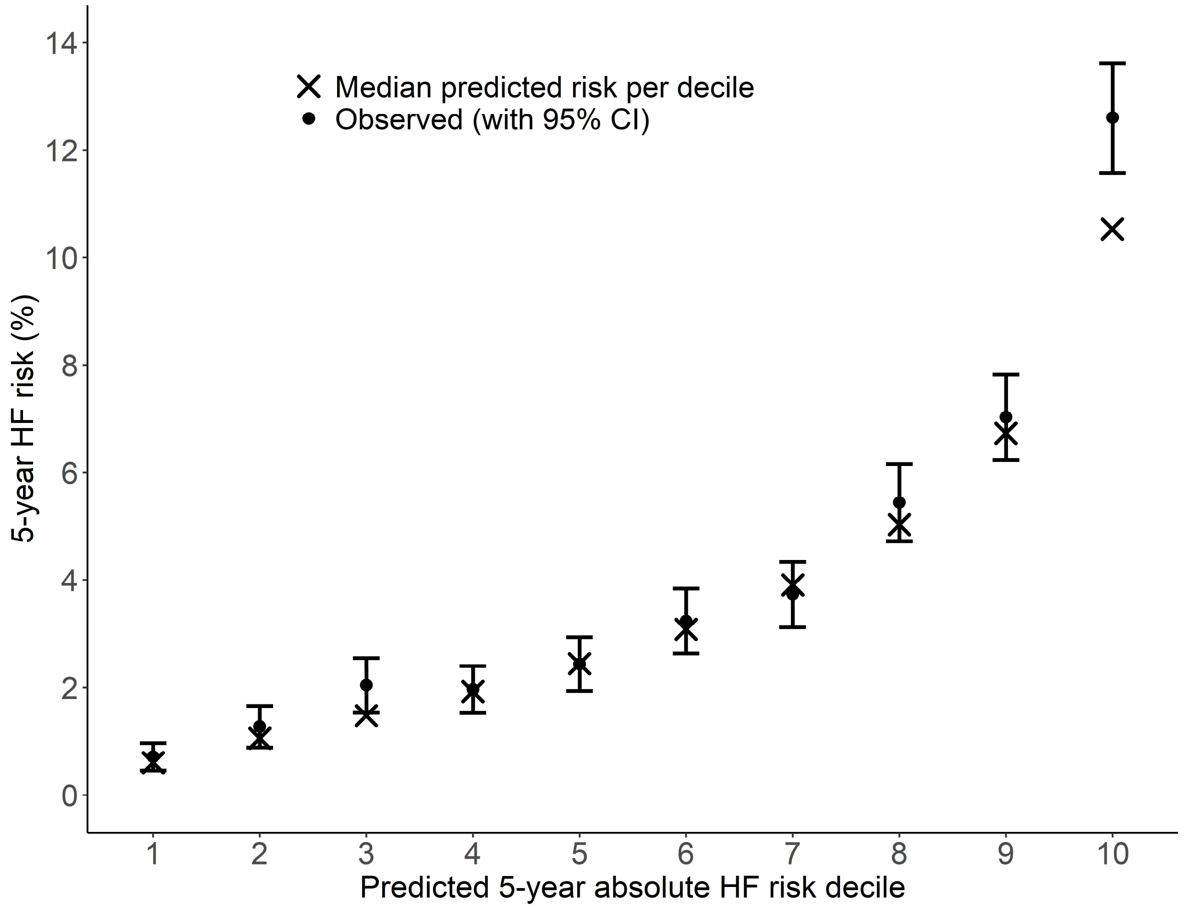

HF=heart failure.

Supplemental Figure 5: Distribution of SABRE-predicted absolute SGLT2-inhibitor benefit on 5-year risk of new onset heart failure in the study cohort (n=169,041).

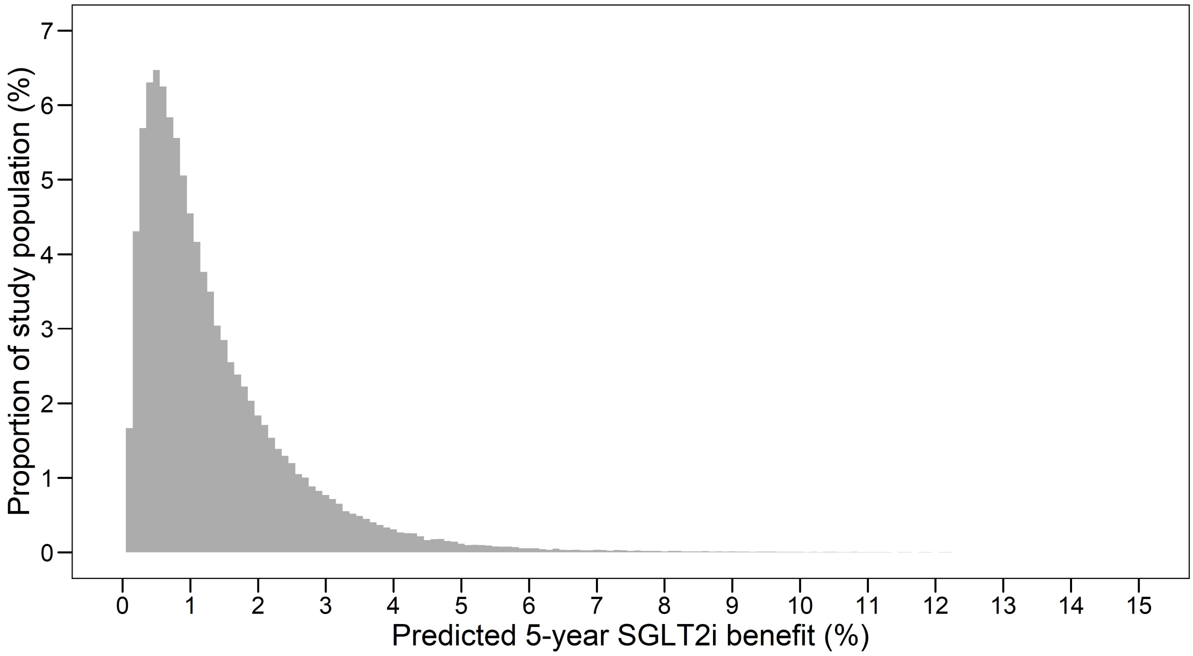

SABRE= SGLT2i absolute benefit response model, SGLT2i=SGLT2i-inhibitor

Supplemental Figure 6: Kaplan-Meier plots of observed heart failure incidence over 5 years for SGLT2-inhibitor treated individuals vs comparator (treated with DPP4-inhibitor/sulfonylurea), with patients stratified based on a 'SABRE model aligned with ADA/EASD' strategy (matched to proportions recommended by ADA/EASD guidance; predicted absolute heart failure benefit >1.0%). Overlap weighting was used within each stratum to balance SGLT2-inhibitor and comparator groups. Observed benefits are estimated differences in survival at 5 years between the weighted SGLT2-inhibitor and comparator groups.

SABRE model aligned with ADA/EASD

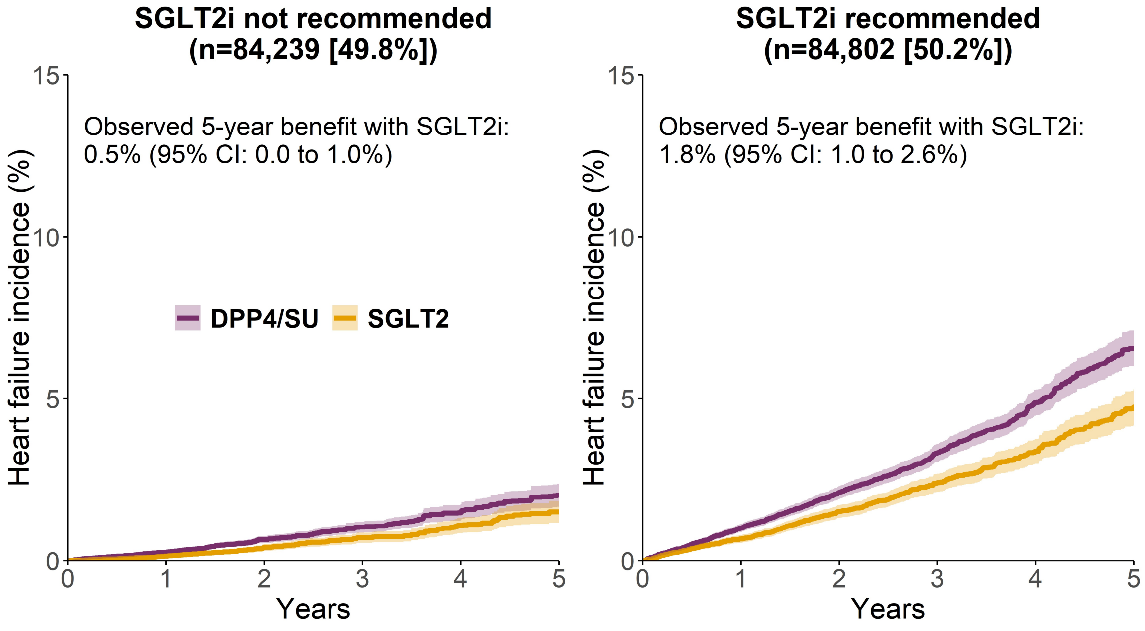

DPP4i=DPP4-inhibitor, SABRE= SGLT2i absolute benefit response model, SGLT2i=SGLT2i-inhibitor, SU=sulfonylurea.

References

1. Rodgers LR, Weedon MN, Henley WE, Hattersley AT, Shields BM. Cohort profile for the MASTERMIND study: using the Clinical Practice Research Datalink (CPRD) to investigate stratification of response to treatment in patients with type 2 diabetes. BMJ Open 2017;7:e017989

Supplemental Appendix 1: STROBE Statement—Checklist of items that should be included in reports of ***cohort studies***

	Item No	Recommendation	Reported in section
**Title and abstract**	1	(*a*) Indicate the study's design with a commonly used term in the title or the abstract	As this is a model development study, we have not described it as a cohort study
		(*b*) Provide in the abstract an informative and balanced summary of what was done and what was found	Abstract
**Introduction**			
Background/rationale	2	Explain the scientific background and rationale for the investigation being reported	Introduction
Objectives	3	State specific objectives, including any prespecified hypotheses	Introduction
**Methods**			
Study design	4	Present key elements of study design early in the paper	Methods
Setting	5	Describe the setting, locations, and relevant dates, including periods of recruitment, exposure, follow-up, and data collection	Methods: study population
Participants	6	(*a*) Give the eligibility criteria, and the sources and methods of selection of participants. Describe methods of follow-up	Methods: study population
		(*b*) For matched studies, give matching criteria and number of exposed and unexposed	N/A
Variables	7	Clearly define all outcomes, exposures, predictors, potential confounders, and effect modifiers. Give diagnostic criteria, if applicable	Methods: study population and outcome and Supplemental Material: extended variable definitions
Data sources/ measurement	8*	For each variable of interest, give sources of data and details of methods of assessment (measurement). Describe comparability of assessment methods if there is more than one group	Methods: study population and Supplemental Material: extended variable definitions
Bias	9	Describe any efforts to address potential sources of bias	Methods: weighting
Study size	10	Explain how the study size was arrived at	Methods: study population

Quantitative variables	11	Explain how quantitative variables were handled in the analyses. If applicable, describe which groupings were chosen and why	Methods: study population and Supplemental Material: extended variable definitions
Statistical methods	12	(*a*) Describe all statistical methods, including those used to control for confounding	Methods: weighting and statistical analysis
		(*b*) Describe any methods used to examine subgroups and interactions	Methods: statistical analysis
		(*c*) Explain how missing data were addressed	Methods: weighting
		(*d*) If applicable, explain how loss to follow-up was addressed	Methods: statistical analysis
		(*e*) Describe any sensitivity analyses	Methods: statistical analysis

Results

Participants	13*	(a) Report numbers of individuals at each stage of study—eg numbers potentially eligible, examined for eligibility, confirmed eligible, included in the study, completing follow-up, and analysed	Results paragraph 1 and Supplemental Figure 1
		(b) Give reasons for non-participation at each stage	Supplemental Figure 1
		(c) Consider use of a flow diagram	Supplemental Figure 1
Descriptive data	14*	(a) Give characteristics of study participants (eg demographic, clinical, social) and information on exposures and potential confounders	Results paragraph 1 and Table 1
		(b) Indicate number of participants with missing data for each variable of interest	Supplemental Figure 1
		(c) Summarise follow-up time (eg, average and total amount)	Results paragraph 1
Outcome data	15*	Report numbers of outcome events or summary measures over time	Results paragraph 2 and Supplemental Figure 3
Main results	16	(*a*) Give unadjusted estimates and, if applicable, confounder-adjusted estimates and their precision (eg, 95% confidence interval). Make clear which confounders were adjusted for and why they were included	Results paragraph 2 and Supplemental Figure 3

		(*b*) Report category boundaries when continuous variables were categorized	N/A
		(*c*) If relevant, consider translating estimates of relative risk into absolute risk for a meaningful time period	Results
Other analyses	17	Report other analyses done—eg analyses of subgroups and interactions, and sensitivity analyses	Results paragraph 2, Figure 1, Supplemental Table 2 and Supplemental Figure 3
**Discussion**			
Key results	18	Summarise key results with reference to study objectives	Discussion: principal findings
Limitations	19	Discuss limitations of the study, taking into account sources of potential bias or imprecision. Discuss both direction and magnitude of any potential bias	Discussion: strengths and weaknesses compared to existing studies and strengths and weaknesses
Interpretation	20	Give a cautious overall interpretation of results considering objectives, limitations, multiplicity of analyses, results from similar studies, and other relevant evidence	Discussion: potential implications for clinicians or policymakers
Generalisability	21	Discuss the generalisability (external validity) of the study results	Discussion: strengths and weaknesses
**Other information**			
Funding	22	Give the source of funding and the role of the funders for the present study and, if applicable, for the original study on which the present article is based	Acknowledgements: funding and assistance

*Give information separately for exposed and unexposed groups.

Note: An Explanation and Elaboration article discusses each checklist item and gives methodological background and published examples of transparent reporting. The STROBE checklist is best used in conjunction with this article (freely available on the Web sites of PLoS Medicine at http://www.plosmedicine.org/, Annals of Internal Medicine at http://www.annals.org/, and Epidemiology at http://www.epidem.com/). Information on the STROBE Initiative is available at http://www.strobe-statement.org.

Supplemental Appendix 2: BePRECISE Checklist: reporting guidelines for precision medicine research

The checklist should be cited as follows: Lim SS, Semnani-Azad Z, Morieri ML, Ng AH, Ahmad A, Fitipaldi H, Boyle J, Collin C, Dennis JM, Langenberg C, Loos RJF, Morrison M, Ramsay M, Sanyal AJ, Sattar N, Hivert MF, Gomez MF, Merino J, Tobias DK, Trenell MI, Rich SS, Sargent JL, Franks PW. *Reporting guidelines for precision medicine research of clinical relevance: the BePRECISE Checklist*. Nature Medicine. 2024.

**Item number**		**Item wording**	**Elaboration and Explanation of Item**	**Reported in this manuscript**
E. Equity, inclusion, diversity and patient and public involvement and engagement **(PPIE)**. Authors are encouraged to address these topics in their manuscripts within relevant sections. The reporting items listed herein are not exhaustive and all considerations of PPIE (including patient-reported outcomes and experience), as well as any community engagement efforts, should be described wherever possible.				
E1		Use appropriate population descriptors such as ancestry, geographic and sociodemographic characteristics of all participants, particularly those in underrepresented groups.	*In cases where data from underrepresented group(s) are collected, and the sub-sample size is n≥20, all data should be analyzed and reported (even in cases where subgroup analyses might be considered underpowered, as this will facilitate subsequent meta-analyses of results). A minimum sample size of 20 is based on the 'All of Us Research Program Data User Code of Conduct' (https://www.researchallofus.org/faq/data-user-code-of-conduct/), and is intended to avoid disclosing individual participant identity.*<br><br>*Avoid merging sub-groups into larger heterogeneous groups (e.g.'non-European ancestry').*<br><br>*While there is ongoing discussion on the appropriate use of words and terms describing groups within populations, this Checklist yields to other guidelines on this matter.* If data pertaining to race and/or ethnicity is collected this should be reported in accordance with relevant established guidance.	Age, sex, ethnicity, deprivation and other sociodemographic characteristics are reported for the full cohort before and after overlap weighting (Supplemental Table 1, Table 1). Ethnicity is self-reported and presented as White, Asian, Black, Mixed/Other and Missing, without aggregation. Ethnicity-stratified hazard ratio estimates are reported where sample size permitted (Supplemental Table 2).

Item number		Item wording	Elaboration and Explanation of Item	Reported in this manuscript
E2		Describe the implications of inclusion and/or exclusion of people who are understudied in precision medicine research or underserved by health services	*Describe implications for successful extrapolation of study findings to other groups, particularly those typically underrepresented in precision medicine research.*	Discussion, Unanswered questions and future research, paragraph 3: *"Finally, further validation of the SABRE model in non-White and older populations will be important to ensure generalisability and equity, particularly given known ethnic and age-related differences in heart failure risk, treatment response, and access to care."*
E3		Describe PPIE in any aspect of the study design, conduct and/or reporting	*PPIE may include consultation, involvement, partnership, or leadership by end-users, including being part of the research and/or authorship team.*	Not reported in main manuscript due to word limitations. However, people with type 2 diabetes were involved in the MASTERMIND consortium and were key in identifying that better, more tailored, evidence was needed for the choice of type 2 diabetes therapy. Preliminary results from this study were presented to the Exeter Diabetes Patient and Public Involvement Group who advised on the interpretation of these results.
E4		Where possible, and ideally with guidance from PPIE representatives, describe the potential impact of the study's results from a lived experience perspective, especially the impact of the research on people living with disease.		Discussion, Potential implications for clinicians or policymakers, paragraph 1: *"The SABRE model demonstrates a novel approach based on individual-level risk estimation to support targeting of SGLT2i within the majority of the T2D population, who do not have ASCVD, HF or CKD, to those who would benefit the most for heart failure prevention."*
**1. Title and/or abstract**				
1.1		Include 'precision medicine' in the title or abstract	*These reporting guidelines use the terms 'precision medicine' and 'personalized medicine', defined elsewhere (Tobias D.K., et al. Nat Med. 2023).*	Manuscript title: *"Precision prescribing of SGLT2-inhibitors in people with type 2 diabetes for primary prevention of heart failure: model development and validation study."*

Item number		Item wording	Elaboration and Explanation of Item	Reported in this manuscript
1.2		State the research question and study design	*‘Study design’ refers to the specific type of clinical trial design (e.g. parallel arm, randomized cross-over, recall-by-genotype) or observational cohort design (e.g. cross-sectional study, prospective cohort study, case-cohort study, case-control study). If the study design involves time-series assessments this should also be highlighted.*	Introduction paragraph 3: *“We aimed to develop and validate an ‘SGLT2i Absolute Benefit REsponse’ model (SABRE) to predict the absolute benefit of SGLT2i for primary prevention of heart failure for individual patients with T2D without ASCVD/HF.”* Methods: study population and treatment comparison detail the study design (observational longitudinal cohort study).
1.3		Describe if the study relates to prevention, diagnostics, treatment and/or prognostics		This study looks at the effect of treatment with SGLT2i on prevention of primary heart failure as described in the Abstract and Introduction.
1.4		Describe population or subgroup that is the focus of the current analysis		Methods, study population: *“The study population was those with type 2 diabetes (T2D), without atherosclerotic cardiovascular disease (ASCVD), heart failure, or chronic kidney disease (CKD; stage 3a–5 or macroalbuminuria), i.e. excluding those for whom evidence-based guidance for SGLT2i treatment already exists.”*

Item number		Item wording	Elaboration and Explanation of Item	Reported in this manuscript
**2. Background and Objectives**				
2.1		State the study hypothesis describing the specific rationale for the precision medicine approach		Rationale: Introduction paragraph 2: *"Beyond these broad recommendations, current guidance does not give advice regarding which people with T2D without ASCVD/HF are likely to have the greatest cardiovascular benefit if treated with SGLT2i."*
2.2		State the study objective(s) of the precision medicine study as either a) etiological, b) discovery, c) predictive and/or d) confirmatory. State all that apply. See the Explanation and elaborations document for detailed descriptions of the objectives.	*a) Etiological: Characterization of heterogeneity across individual-level data*<br>*b) Discovery: Exploration of associations between a set of clinical features and outcome heterogeneity (e.g. descriptive RCT subgroup analysis or exploratory analysis of risk factors)*<br>*c) Predictive: Development of a specific approach(es) to predict heterogeneity in clinical or treatment-related outcomes for individuals or subgroups*<br>*d) Confirmatory: Reproduction of a previously proposed precision medicine approach*	c) Predictive: Introduction paragraph 3: *"We aimed to develop and validate an 'SGLT2i Absolute Benefit REsponse' model (SABRE) to predict the absolute benefit of SGLT2i for primary prevention of heart failure for individual patients with T2D without ASCVD/HF."*
**3. Methods**				
3.1		Describe aspects of the study design relevant to precision medicine that are necessary for the design to be adequately understood by the reader.		Methods: study population and treatment comparison fully detail the study design (observational longitudinal cohort study).

Item number		Item wording	Elaboration and Explanation of Item	Reported in this manuscript
3.2		Provide the rationale for choice of outcome(s).		Introduction paragraph 1: *"SGLT2-inhibitors (SGLT2i) substantially reduce the risk of hospitalisation for heart failure and, to a lesser extent, reduce atherosclerotic cardiovascular disease (ASCVD) events in people with type 2 diabetes (T2D)"*
3.3		If the dataset is a subset of a larger study, describe how and why the subset(s) of participants used in the analysis was selected.		Not applicable; the analysis used the full eligible study population defined by prespecified inclusion and exclusion criteria.
3.4		Define any markers used for stratification or prediction of outcomes in individuals or subgroups	*'Markers' in this context could include (and are not limited to) biomarkers, molecular markers and clinical characteristics, as well as societal, economic, geographic, and cultural factors.*	Sensitivity analysis of hazard ratio estimates for SGLT2i vs comparator was carried out in sex- and ethnicity-defined subgroups (Methods, Statistical analysis, Sensitivity analyses and Supplemental Table 3).
3.5		Provide details of any measures taken to mitigate type 1 and/or type 2 error. Describe *a priori* power calculations and adjustment for multiple-testing, if performed.		Overlap weighting (Methods, Weighting) and prespecified modelling approaches (Methods, Statistical analysis) were used.
3.6		Describe any approach used for internal and/or external replication and/or validation and whether these analyses were planned, and relevant datasets identified before or after conclusion of primary analyses.	*'Replication' analyses are those that seek to directly reproduce primary analyses. 'Validation' analyses are those that seek to generate results using orthogonal methods to those used in the primary analyses that strengthen its conclusions.*	Internal validation of heart failure risk predictions and SGLT2i benefit predictions were carried out as prespecified in Methods, Statistical analysis.

Item number		Item wording	Elaboration and Explanation of Item	Reported in this manuscript
3.7		Specify how the sample size for any replication/validation study was determined		Sample size calculations were not required as the study population was sufficiently large (with sufficiently large numbers of outcome events in both treatment arms for the primary analysis) for this study design.
**4. Results**				
4.1		Specify the number of participants in each analysis and provide baseline characteristics	*If analysis includes comparison of subgroups, baseline characteristics for each subgroup should be provided.*	Reported in Supplemental Table 1 (before weighting) and Table 1 (after weighting).
4.2		Report statistical tests and results for subgroup comparisons.	*Comparisons between subgroups should include appropriate test statistics, which may include tests of interaction and heterogeneity, and in cluster analyses tests of probability for cluster assignment (e.g., relative entropy statistic).*	Heterogeneity of SGLT2i benefit by baseline heart failure risk was analysed as per Methods, Statistical analysis, Evaluation of accuracy of SABRE model inputs: *"a Cox model extended to incorporate a drug (SGLT2i versus DPP4i/SU) by QDiabetes-HF risk score (3-knot restricted cubic spline) interaction term."* The results are reported in Figure 1 and in Results paragraph 2.
4.3		If benchmarking against current practice was undertaken, describe these results. State if benchmarking was not performed	*Provide formal comparisons against current practice to assess performance of the precision medicine approach. For example, for prediction models, compare new biomarkers with established prediction variables, formally testing differences in prediction performance. For treatments, compare measures of clinical effectiveness (e.g. number needed to treat) between new and conventional approaches. If such comparisons are not possible, provide an explanation.*	Comparisons to other treatment strategies have been performed using absolute benefit (primary HF events prevented per 100 patient-years of treatment in those treated) and number needed to treat per year to prevent one HF event (Figure 3).

Item number		Item wording	Elaboration and Explanation of Item	
4.4		Provide results for all attempted validation and/or replication analyses		Validation results are reported in Supplemental Figure 4 (heart failure risk predictions) and Figure 2 (SGLT2i benefit predictions).
**5. Discussion**				
5.1		General limitations	*Describe how study characteristics or analytical methods may introduce bias, particularly as these pertain to features of the analysis related to precision medicine (e.g., subgroup comparisons)*	Discussion of limitations of analytical method (observational study design) and study characteristics (small numbers of non-White ethnicity, all aged≤84 years, only routine clinical features available) are included in the Discussion, Strengths and weaknesses.
5.2		Interpretation: Describe the precision medicine approach that could potentially be applied in clinical practice		Discussion, Potential implications for clinicians or policymakers: *"The SABRE model demonstrates a novel approach based on individual-level risk estimation to support targeting of SGLT2i within the majority of the T2D population, who do not have ASCVD, HF or CKD, to those who would benefit the most for heart failure prevention."*
